# Relationship between plasma growth differentiation factor-15 levels and diabetic retinopathy in individuals with type 2 diabetes

**DOI:** 10.1038/s41598-020-77584-z

**Published:** 2020-11-25

**Authors:** Jin Ook Chung, Seon-Young Park, Dong Hyeok Cho, Dong Jin Chung, Min Young Chung

**Affiliations:** 1 Division of Endocrinology and Metabolism, Department of Internal Medicine, Chonnam National University Medical School, 8 Hak-Dong, Dong-Gu, Gwangju, 501-757 Republic of Korea; 2 Division of Gastroenterology and Hepatology, Department of Internal Medicine, Chonnam National University Medical School, 8 Hak-Dong, Dong-Gu, Gwangju, 501-757 Republic of Korea

**Keywords:** Endocrinology, Endocrine system and metabolic diseases

## Abstract

The purpose of our study was to investigate the relationship between plasma growth differentiation factor-15 (GDF-15) concentrations and diabetic retinopathy in patients with type 2 diabetes mellitus (DM). We evaluated 235 patients with type 2 DM in a cross-sectional study. Significantly increased levels of the plasma GDF-15 were found in individuals with diabetic retinopathy versus those without. According to the degree of diabetic retinopathy, there was a significant difference in the average plasma GDF-15 levels (no diabetic retinopathy, 1114 ng/L; nonproliferative diabetic retinopathy, 1327 ng/L; proliferative diabetic retinopathy, 1445 ng/L; *p* for trend = 0.035) after adjustments for confounders. Logistic regression analyses indicated that plasma GDF-15 concentrations were significantly associated with diabetic retinopathy (odds ratio per 1 standard deviation increment in the log-transformed value, 1.78; 95% confidence interval, 1.05–3.03, *p* = 0.032). Our study showed a significant positive relationship between plasma GDF-15 concentrations and diabetic retinopathy in type 2 DM patients.

## Introduction

Diabetic retinopathy is a common manifestation of diabetes-related microvascular damage in the retina, which can result in blindness in patients with diabetes mellitus (DM)^1^. Apart from the deleterious effect on vision, many studies have shown that diabetic retinopathy is linked to increased risks of systemic vascular diseases and mortality in type 2 DM patients^1^. In addition, diabetic retinopathy may result in a significant reduction in functional status and a decline in the quality of life^2^. Despite many studies demonstrating that diabetic retinopathy is primarily driven by hyperglycemia, it is unlikely that the risk of diabetic retinopathy can be accounted for by a single factor, indicating that its pathogenesis is multifactorial^1^.

Growth differentiation factor-15 (GDF-15) belongs to the transforming growth factor-β family and is also known as macrophage inhibitory cytokine-1. Its expression is enhanced in response to tissue ischemia^3^. Many previous studies have demonstrated that elevated GDF-15 concentrations were positively related with cardiovascular injury and mortality in humans^4,5^, suggesting its role as a novel biomarker for cardiovascular diseases^6^. Previous investigations have also shown a positive association between GDF-15 concentrations and diabetes risk^6,7^. Moreover, GDF-15 is reported to be implicated in the retinal inflammatory response^8^. However, the association GDF-15 levels and diabetic retinopathy in type 2 DM patients is not clear. Therefore, investigating whether GDF15 is implicated in the risk of diabetic retinopathy in type 2 DM patients is warranted.

The purpose of our study was to examine the association between plasma GDF-15 concentrations and diabetic retinopathy in individuals with type 2 DM.

## Results

Table 1 presents the characteristics of the participants. The patients with diabetic retinopathy had a higher prevalence of hypertension, lower BMIs, higher HbA1c levels, a longer duration of DM, lower eGFRs, and higher AERs than those without. The patients with diabetic retinopathy more frequently received insulin treatment compared with those without. Among those with diabetic retinopathy, the prevalence of hypertension was greater and the plasma GDF-15 concentrations were significantly higher (Table 1).

**Table 1:** Characteristics of patients with type 2 DM according to diabetic retinopathy.

	DR (–)	NPDR	PDR	*P*-value	DR (+)	*p*-value DR (+) vs. DR (–)
n	168	47	20		67	
Men, n (%)	78 (46.4)	27 (57.4)	5 (25.0)	0.051	32 (47.8)	0.853
Age (years)	59.4 ± 12.2	63.6 ± 12.3	60.2 ± 12.0	0.115	62.6 ± 12.2	0.071
Diabetes duration (years)	2.0 (0.2–10.0)	12.0 (7.0–22.0)	16.5 (3.3–23.8)	< 0.001	13.0 (5.0–22.0)	< 0.001
Body mass index (kg/m^2^)	26.2 ± 3.8	25.0 ± 4.3	24.4 ± 2.8	0.045	24.8 ± 3.9	0.015
Systolic BP (mmHg)	133.3 ± 18.2	133.8 ± 15.5	141.2 ± 22.4	0.186	136.0 ± 18.0	0.299
Diastolic BP (mmHg)	77.7 ± 12.8	75.1 ± 12.9	78.2 ± 14.7	0.450	76.0 ± 13.5	0.373
Hypertension, n (%)	87 (51.8)	31 (66.0)	16 (80.0)	0.021	47 (70.1)	0.010
Hyperlipidemia, n (%)	96 (57.1)	24 (51.1)	12 (60.0)	0.711	36 (53.7)	0.634
HbA_1c_ (mmol/moL)	59 ± 20	67 ± 18	62 ± 16	0.044	66 ± 17	0.022
HbA_1c_ (%)	7.6 ± 1.8	8.3 ± 1.6	7.8 ± 1.4	0.044	8.2 ± 1.6	0.022
Triglyceride (mmol/L)	1.3 (1.0–1.8)	1.2 (1.0–1.9)	1.3 (1.1–2.1)	0.753	1.3 (1.0–1.9)	0.964
Total cholesterol (mmol/L)	4.4 ± 1.2	4.2 ± 1.3	4.3 ± 1.3	0.266	4.2 ± 1.3	0.126
LDL-cholesterol (mmol/L)	2.6 ± 0.9	2.5 ± 0.9	2.6 ± 1.0	0.312	2.5 ± 0.9	0.241
HDL-cholesterol (mmol/L)	1.3 ± 0.3	1.2 ± 0.3	1.1 ± 0.3	0.153	1.2 ± 0.3	0.085
hs-CRP (mg/dL)	0.16 (0.10–0.22)	0.20 (0.09–0.32)	0.15 (0.03–0.33)	0.808	0.18 (0.09–0.28)	0.659
GDF-15 (ng/L)	974 (701–1466)	1535 (981–2305)	1839 (1004–2484)	< 0.001	1535 (995–2455)	< 0.001
eGFR (mL∙min^−^^1^1.73 m^−2^)	94.1 ± 17.6	84.6 ± 23.8	88.1 ± 12.9	0.009	85.6 ± 23.1	0.008
AER (mg/gCr)	12.2 (6.8–31.5)	21.3 (10.1–58.9)	38.6 (16.7–260.5)	< 0.001	23.0 (12.2–71.9)	< 0.001
Use of insulin, n (%)	10 (6.0)	11 (23.4)	8 (40.0)	< 0.001	19 (28.4)	< 0.001
Use of OHAs, n (%)	107 (63.7)	31 (66.0)	12 (60.0)	0.896	43 (64.2)	0.944

The values are expressed as mean ± standard deviation or median (interquartile range) unless otherwise noted.

AER, albumin excretion rate; BP, blood pressure; DM, diabetes mellitus; DR, diabetic retinopathy; eGFR, estimated glomerular filtration rate; GDF-15, growth differentiation factor-15; HbA_1c_, glycated hemoglobin; HDL–cholesterol, high density lipoprotein cholesterol; hs-CRP, high-sensitivity C-reactive protein; LDL–cholesterol, low density lipoprotein cholesterol; NPDR, nonproliferative diabetic retinopathy; OHAs, oral hypoglycemic agents; PDR, proliferative diabetic retinopathy.

Table 2 displays the average (95% confidence interval [CI]) plasma GDF-15 concentrations based on the degree of diabetic retinopathy. After adjusting for sex, age, hyperlipidemia, hypertension, hs-CRP, BMI, HbA_1c_, DM duration, eGFR, AER, and the use of insulin and OHAs, there was a significant difference in the average GDF-15 plasma levels according to the degree of diabetic retinopathy (no diabetic retinopathy, 1114 ng/L, 95% CI: 1033–1199; NPDR, 1327 ng/L, 95% CI: 1143–1538; PDR, 1445 ng/L, 95% CI: 1161–1803; *p* for trend = 0.035).

**Table 2:** Average plasma GDF-15 levels according to diabetic retinopathy severity in patients with type 2 DM.

	Severity	Model 1	Model 2	Model 3
		Mean (95% CI)	Mean (95% CI)	Mean (95% CI)
Plasma GDF-15 (ng/L)^†^	Normal	1028 (948–1114)	1045 (959–1135)	1114 (1033–1199)
	NPDR	1442 (1236–1683)	1556 (1324–1828)	1327 (1143–1538)
	PDR	1603 (1268–2032)	1710 (1337–2193)	1445 (1161–1803)
	*p* for trend	< 0.001	< 0.001	0.035

The data are shown as means (95% CI). ^†^Values were log-transformed prior to analyses.

Model 1: adjusted by sex and age.

Model 2: model 1 + adjusted by BMI, hs-CRP^†^, hyperlipidemia, and hypertension.

Model 3: model 2 + adjusted by plus HbA_1c_, diabetes duration^†^, AER^†^, eGFR, and use of insulin and OHAs.

AER, albumin excretion rate; BMI, body mass index; CI, confidence interval; DM, diabetes mellitus; eGFR, estimated glomerular filtration rate; GDF-15, growth differentiation factor-15; HbA_1c_, glycated hemoglobin; hs-CRP, high-sensitivity C-reactive protein; OHAs, oral hypoglycemic agents.

As shown in Table 3, we conducted logistic regression analysis to assess the effects of GDF-15 concentrations on diabetic retinopathy. After adjustment for sex, age, hyperlipidemia, hypertension, hs-CRP, BMI, HbA1c, DM duration, eGFR, AER, and the use of insulin and OHAs, a statistically significant association was persistent between plasma GDF-15 concentrations and diabetic retinopathy (OR per 1 SD increment in the log-transformed value, 1.78; 95% CI: 1.05–3.03, *p* = 0.032).

**Table 3:** Logistic regression models for the association between GDF-15 levels and diabetic retinopathy in patients with type 2 DM.

	Unadjusted			Adjusted*		
	OR	95% CI	*p*-value	OR	95% CI	*p*-value
GDF-15 (ng/L)^†^	2.25	1.58–3.21	< 0.001	1.78	1.05–3.03	0.032
BMI (kg/m^2^)	0.91	0.83–0.98	0.016	0.94	0.85–1.05	0.279
Hypertension (yes)	2.19	1.20–4.01	0.011	1.72	0.72–4.10	0.219
HbA_1c_ (%)	1.20	1.02–1.40	0.024	1.02	0.81–1.27	0.877
Diabetes duration (years)^†^	8.36	4.09–17.08	< 0.001	6.06	2.18–16.88	0.001
AER (mg/gCr)^†^	2.72	1.66–4.43	< 0.001	1.68	0.90–3.14	0.102
eGFR (mL∙min^−1^1.73 m^−2^)	0.98	0.96–0.99	0.003	0.99	0.97–1.02	0.565
Use of insulin (yes)	6.25	2.72–14.36	< 0.001	2.48	0.42–14.54	0.314

^†^Values were log-transformed prior to analyses.

Adjusted for age, sex, hyperlipidemia, hs-CRP^†^, and use of OHAs.

AER, albumin excretion rate; BMI, body mass index; CI, confidence interval; DM, diabetes mellitus; eGFR, estimated glomerular filtration rate; GDF-15, growth differentiation factor-15; HbA_1c_, glycated hemoglobin; hs-CRP, high-sensitivity C-reactive protein; OHAs, oral hypoglycemic agents; OR, Odds ratio.

**Figure 1:**
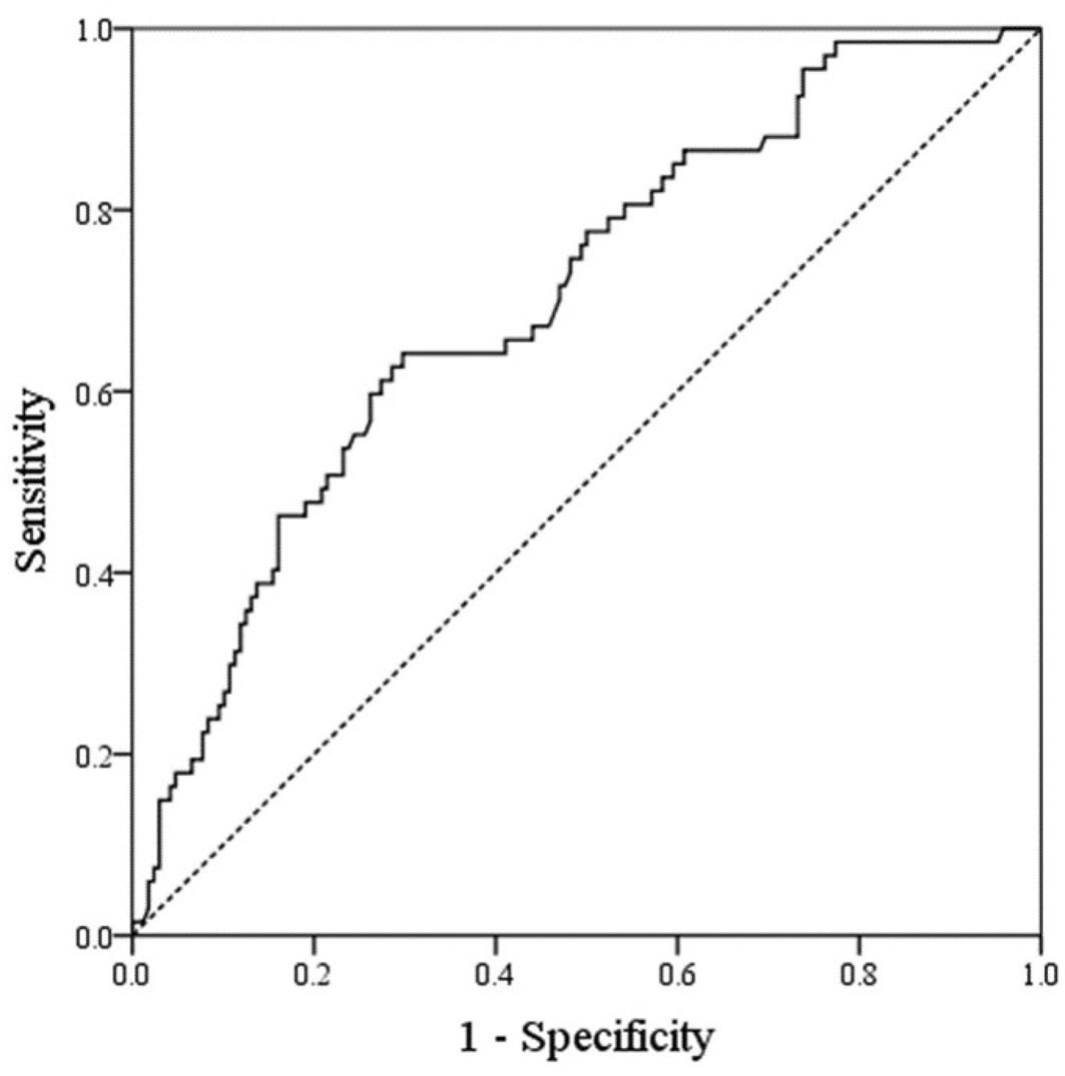
ROC analysis of GDF-15 levels in predicting diabetic retinopathy in individuals with type 2 diabetes. AUC = 0.701 (*p* < 0.001), 95% CI: 0.629–0.774. Identified GDF-15 cutoff value = 1336 ng/L; sensitivity: 64.2%; specificity: 70.2%. ROC, receiver operating characteristic; AUC, area under the curve, GDF-15, growth differentiation factor-15.

Analysis of the ROC curve of GDF-15 is displayed in Fig. 1. The AUC for GDF-15 was 0.701 (95% CI: 0.629–0.774; *p* < 0.001), and the optimal cut-off value was 1336 ng/L with 70.2% specificity and 64.2% sensitivity in predicting diabetic retinopathy.

## Discussion

Our study found that plasma GDF-15 concentrations were independently and positively associated with diabetic retinopathy in individuals with type 2 DM. Our data revealed that the severity of diabetic retinopathy might be related with plasma GDF-15 concentrations. In addition, our findings suggest that GDF-15 may be a valuable biomarker for discriminating diabetic retinopathy in patients with type 2 DM.

GDF-15 has been increasingly recognized as a stress response cytokine^3^, and suggested to play a role in cell growth, differentiation, and inflammation^3^. GDF-15 is secreted from various cells, including endothelial cells and macrophages after tissue injury^6^, and increased GDF-15 levels may indicate tissue damage^9^. Previous studies emphasizing the association between plasma GDF-15 concentrations and cardiovascular disease have suggested GDF-15 as a potential biomarker for cardiovascular diseases. This was verified by numerous investigations demonstrating that high circulating levels of GDF-15 were related to the increased risks of heart failure, stroke, coronary heart disease, and cardiovascular mortality^4,10,11^.

Close associations between GDF-15 and increased risk of diabetes have been recently reported ^6,9,12^. Kempf et al.^12^ showed a positive correlation between plasma GDF-15 levels and insulin resistance. In addition, previous clinical investigations showed that GDF-15 levels were higher in individuals with type 2 DM compared with those without type 2 DM^13,14^. In a study with obese women, Dostálová et al.^7^ found that serum GDF-15 concentrations were increased in women with type 2 DM compared with control participants. Recently, Bao et al.^15^ reported that higher levels of GDF-15 were associated with increased incidence of diabetes. In addition, GDF-15 might be implicated in retinal inflammation and injury^8,16^. In response to optic nerve injury, the GDF-15 levels in the retina might be upregulated, as shown in previous research^16^. Ilhan et al.^8^ reported that increased levels of GDF-15 in the vitreous were found in inflammatory vitreoretinal disorders. However, the relationship between plasma GDF-15 concentrations and diabetic retinopathy in type 2 DM patients is not clear. Our data indicate a close association between GDF-15 levels and diabetic retinopathy in type 2 DM patients. We observed that there were significant differences in DM duration and HbA1c levels between patients with diabetic retinopathy and those without. Diabetic retinopathy was also related to impaired renal function and hypertension, consistent with previous reports^1,17^. Moreover, previous investigations have shown that GDF-15 levels were related to HbA1c levels^4,6^, and have reported that increased levels of GDF-15 were associated with renal impairment and increased prevalence of hypertension^4,6^. Hence, it is presumed that these might be partially responsible for the observed relationship between GDF-15 and retinopathy. However, our multivariable analysis showed that the statistically significant relationship between GDF-15 and diabetic retinopathy persisted despite adjustment for confounders including hypertension, HbA1c, DM duration, eGFR, and AER (Table 3). Consequently, the findings indicate that those variables did not exert significant influences on the relationship between them.

Even though the mechanisms by which GDF-15 might be implicated in the pathogenesis of diabetic retinopathy is not clear, possible explanations have been suggested. GDF-15 is involved in oxidative stress and endothelial function^6,18^. GDF-15 is also implicated in inflammatory and immune processes^19,20^. These are key pathogenic pathways of diabetic retinopathy^1^. Consequently, the close relationship between GDF-15 and diabetic retinopathy may be explained by the common pathways involved, while the causal inferences could not be drawn in our study. Further research is required to investigate the exact underlying mechanisms.

In the present study, we also found a positive association between plasma GDF-15 levels and the severity of diabetic retinopathy. Even though so far there are no available clinical data on the relationship between GDF-15 and pathogenesis of diabetic retinopathy in patients with type 2 DM, a possible implication of GDF-15 in progression of diabetic microvascular injury has been suggested. Hellemons et al.^9^ reported that increased circulating levels of GDF-15 were related with transition from normo- to micro- and from micro- to macroalbuminuria in patients with type 2 DM. Thus, large longitudinal studies are necessary to verify the usefulness of GDF-15 in prediction of retinal disease progression.

CRP is a well-recognized marker of systemic inflammation^21^. Despite extensive evidence indicating a link between inflammation and diabetic retinopathy^1,22^, the data in the literature in regard to the association between CRP and retinopathy are controversial^23,24^. In addition, in our study, multivariable analysis showed that the statistically significant relationship between GDF-15 levels and diabetic retinopathy persisted despite adjustment for confounders including hs-CRP levels. As a consequence, our findings suggest that GDF-15 might be linked to diabetic retinopathy through mechanisms independent of CRP. However, further research is necessary as other factors besides inflammatory stimuli can influence CRP levels^25^.

This study has some limitations. Due to a cross-sectional nature of the study, the causal relationship could not be established. Because of the relatively small sample size in this study, we were unable to perform further analysis on the differences between individual grades of NPDR. In addition, the number of patients with diabetic macular edema observed in our study was small (< 10%); hence, we could not conduct an analysis on diabetic macula edema. In spite of these limitations, our data might provide a valuable information on associations between GDF-15 levels and diabetic retinopathy in patients with type 2 DM.

In conclusion, plasma GDF-15 concentrations were positively associated with diabetic retinopathy in patients with type 2 DM. It is necessary to explore the underlying mechanisms between GDF-15 and retinopathy in individuals with type 2 DM in the future research.

## Methods

### Participants

In the current cross-sectional study, we studied consecutively enrolled 235 patients with type 2 DM attending the diabetes clinic of our hospital. According to the Report of the Expert Committee on the Diagnosis and Classification of Diabetes Mellitus^26^, type 2 DM was diagnosed. Patients using glucocorticoids, those with renal impairment (serum creatinine ≥ 177 µmol/L), infections or inflammatory disorders, chronic liver disease, occlusive peripheral artery disease, stroke, cardiac disease, or malignancies were excluded from the study. Clinical information regarding past medical history and diabetes duration was obtained through a standardized inquiry. Hypertension was defined as taking anti-hypertensive medications or blood pressure ≥ 140/90 mmHg. Hyperlipidemia was considered as the use of lipid-lowering medications, total serum cholesterol concentrations ≥ 6.5 mmol/L, or triglyceride concentrations ≥ 2.3 mmol/L. The protocol was approved by the ethics committee of Chonnam National University Hospital, and all participants provided informed consent. The study was performed according to the Helsinki Declaration-based ethical principles for medical research involving human subjects.

### Measurements

After the patients fasted overnight, venous blood samples were collected. Ion-exchange liquid chromatography (Tosoh, Tokyo, Japan) was used to assay glycated hemoglobin (HbA1c) levels. We measured high-sensitivity C-reactive protein (hs-CRP) levels by an immunonephelometric assay (Dade Behring, Marburg, Germany). The plasma GDF-15 concentrations were measured using a Human GDF-15 Quantikine enzyme-linked immunosorbent assay (R&D Systems, Minneapolis, MN, USA). The intra-assay and inter-assay variabilities were < 5.0% and < 8.0%, respectively. Using the equation from the Chronic Kidney Disease Epidemiology Collaboration^27^, we assessed the estimated glomerular filtration rate (eGFR). We determined the urinary albumin excretion rate (AER) using the urinary albumin-to-creatinine ratio in random urine samples. An ophthalmologist carried out a dilated fundus examination to evaluate diabetic retinopathy. The patients were classified into three groups: no diabetic retinopathy, nonproliferative diabetic retinopathy (NPDR), and proliferative diabetic retinopathy (PDR). Diabetic retinopathy referred to NPDR or PDR in the current study. Intra-grader reliability was assessed for 50 randomly chosen cases by re-classifying the retinal photographs. The intra-grader reliability kappa value was 0.91, indicating good reliability.

### Statistical analyses

Sample size was determined to detect a medium effect size (d) of 0.5 with an alpha of 0.05 and 90% power. By using G*Power 3.1.9.2^28^, this sample size was calculated for a 1:2 ratio of retinopathy/no retinopathy using two-tailed test. The estimated sample size was 192.

Statistical analysis was done using the Statistical Package for the Social Sciences software version 20.0 (SPSS, Chicago, IL, USA). The means ± standard deviation (SD) and frequencies (percentages) for the variables are presented unless otherwise described. Using the Kolmogorov–Smirnov test, we assessed the normal distribution of each variable. Differences in categorical and continuous variables for individuals with and without diabetic retinopathy were compared using chi-squared test, the Student’s t-test, or Mann–Whitney U test when appropriate. Differences among three groups were tested using analysis of variance or Kruskal–Wallis test. In order to compare the average GDF-15 concentrations according to the degree of diabetic retinopathy, we performed an analysis of covariance after adjustments for other covariates. For variables with skewed distributions, we performed logarithmic transformation before the analyses. To investigate the association between the GDF-15 concentrations and diabetic retinopathy, we carried out multiple logistic regression analyses using independent variables and factors with independent associations. Since the GDF-15 levels were not normally distributed, the odds ratios (ORs) per 1 SD increase in the log-transformed value of GDF-15 were calculated. A dummy variable was used to code the use of insulin and oral hypoglycemic agents (OHAs). In the fully adjusted regression model, age, sex, body mass index (BMI), hs-CRP, hyperlipidemia, hypertension, HbA_1c_, DM duration, AER, eGFR, and use of insulin and OHAs were included as covariates. A receiver operating characteristic (ROC) curve was generated to evaluate the specificity, sensitivity, and area under the curve (AUC) for predicting diabetic retinopathy. To determine the optimal cut-off values, the Youden index was estimated. A *p-*value of < 0.05 indicated statistical significance.
